# Temporal Dynamics of Subjective and Objective Alertness During Exposure to Bright Light in the Afternoon for 5 h

**DOI:** 10.3389/fphys.2021.771605

**Published:** 2021-12-07

**Authors:** Xue Luo, Taotao Ru, Qingwei Chen, Fan-Chi Hsiao, Ching-Sui Hung, Chien-Ming Yang, Guofu Zhou

**Affiliations:** ^1^School of Psychology, South China Normal University, Guangzhou, China; ^2^Lab of Light and Physiopsychological Health, National Center for International Research on Green Optoelectronics, South China Normal University, Guangzhou, China; ^3^Guangdong Provincial Key Laboratory of Optical Information Materials and Technology and Institute of Electronic Paper Displays, South China Normal University, South China Academy of Advanced Optoelectronics, Guangzhou, China; ^4^Department of Counseling and Industrial/Organizational Psychology, Ming Chuan University, Taoyuan, Taiwan; ^5^Department of Psychology, National Chengchi University, Taipei, Taiwan; ^6^The Research Center for Mind, Brain, and Learning, National Chengchi University, Taipei, Taiwan

**Keywords:** light, illumination, alertness, light exposure, electroencephalogram

## Abstract

Light can induce an alertness response in humans. The effects of exposure to bright light vs. dim light on the levels of alertness during the day, especially in the afternoon, as reported in the literature, are inconsistent. This study employed a multiple measurement strategy to explore the temporal variations in the effects of exposure to bright light vs. regular office light (1,200 lx vs. 200 lx at eye level, 6,500 K) on the alertness of participants for 5 h in the afternoon. In this study, 20 healthy adults (11 female; mean age 23.25 ± 2.3 years) underwent the Karolinska sleepiness scale (KSS), the auditory psychomotor vigilance test (PVT), and the waking electroencephalogram (EEG) test for two levels of light intervention. The results yielded a relatively lower relative delta power and a relatively higher beta power for the 1,200 lx condition in comparison with the 200 lx condition. However, the light conditions elicited no statistically significant differences in the KSS scores and performance with respect to the PVT. The results suggested that exposure to bright light for 5 h in the afternoon could enhance physiological arousal while exerting insignificant effects on subjective feelings and performance abilities relating to the alertness of the participants.

## Introduction

Since the discovery of intrinsically photosensitive retinal ganglion cells (ipRGCs), light is shown to have an influence on human physiological and psychological functions as a result of the so-called non-image forming (NIF) functions of light (Berson et al., 2002; Hattar et al., 2002), such as the effects of light on alertness (Cajochen, 2007; Figueiro et al., 2018; Pachito et al., 2018; Souman et al., 2018; Xu and Lang, 2018), mood (Bedrosian and Nelson, 2013, 2017), cognitive function (Vandewalle et al., 2009; Fisk et al., 2018; Ru et al., 2019, 2021), and the regulation of the circadian rhythm (LeGates et al., 2014; Prayag et al., 2019b; Tahkamo et al., 2019) and sleep (Figueiro et al., 2017; Boubekri et al., 2020; Peeters et al., 2021; Stefani et al., 2021). Alertness is a key topic of such research due to its importance to human life. Although the effects of light on alertness have been explored using both subjective and objective measures, the research results are far from consistent, especially the results for diurnal studies (Lok et al., 2018a; Souman et al., 2018; Xu and Lang, 2018; Sithravel and Ibrahim, 2021). Several factors may contribute to the discrepancies in research, such as the light level and the spectrum, the time of day/year, the duration of light exposure, as well as previous light history before the laboratory studies commenced. Optimization of the daytime functions of office workers with respect to artificial light is a primary concern in the interior design and ergonomics of buildings. The current literature suggests that manipulating the level of office light rather than correlation of the color temperature (CCT) can elicit greater non-visual effects during the daytime (Min et al., 2013; Park et al., 2013; Ru et al., 2019). Therefore, the current study is concerned with how high-intensity light influences the physiological and psychological alertness of participants during the daytime.

While the effects of the intensity of polychromatic white light on alertness during the daytime have been extensively studied, the results have revealed no conclusive or consistent findings based on subjective ratings, performance, and physiological indicators of alertness (Lok et al., 2018a; Souman et al., 2018). Some studies have reported positive effects of bright light vs. dim light on the subjective ratings of alertness (Akerstedt et al., 2003; Phipps-Nelson et al., 2003; Ruger et al., 2006; Vandewalle et al., 2006; Iskra-Golec and Smith, 2008; Smolders et al., 2012; Smolders and De Kort, 2014; Huiberts et al., 2017; Te Kulve et al., 2017), while others have reported no significant effects (Sahin et al., 2014; Huiberts et al., 2016). Additionally, the same situation applies regarding the effects of light levels on objective alertness as assessed by undertaking computerized tasks [e.g., the psychomotor vigilance test (PVT)] (Phipps-Nelson et al., 2003; Smolders et al., 2012; Smolders and De Kort, 2014; Huiberts et al., 2016, 2017; Maierova et al., 2016; Münch et al., 2016; Te Kulve et al., 2017), during which the duration of light exposure ranged from 15 min to 5 h. In addition, exposure to bright light vs. dim light is shown to moderate physical arousal as measured by the electroencephalogram (EEG), suggesting a decrease in lower power densities, such as the theta and the theta–alpha band (Vandewalle et al., 2006; Sahin et al., 2014; Smolders et al., 2016). These findings suggested that the alertness effects of light vary as a function of the waveband of the light for different areas of the brain (Smolders et al., 2016). For instance, the regulation of the theta wave is most prominent in the frontal area of the brain, which is associated with sleepiness and the homeostatic sleep drive (Cajochen et al., 2001; Strijkstra et al., 2003). Recently, two independent studies explored the dose-response relationships between the light level and alertness during the daytime (Lok et al., 2018b; Smolders et al., 2018). Unlike the dose-response relationships established during the nighttime (Cajochen et al., 2000), no conclusive findings were found for these two diurnal studies. It is speculated that the relatively short duration (about 1 h) of light exposure in these studies might have yielded results that were not conclusive (Smolders et al., 2018). Thus, whether the duration of light exposure would moderate the alertness effects in response to daytime light remains largely unknown.

The duration of light exposure has been considered a potential variable in moderating the NIF effects of daytime light on mental function (Khademagha et al., 2016; Lok et al., 2018a; Souman et al., 2018; Sithravel and Ibrahim, 2021). The time durations adopted in the previous studies ranged from 15 min (Iskra-Golec and Smith, 2008) to 10 h (Maierova et al., 2016). Indeed, the duration of light exposure was less than 1 h in 10 of 19 studies reviewed in a recent review article (Lok et al., 2018b). Although an exposure duration as short as 1 min has been shown to have significant NIF effects during the nighttime (Prayag et al., 2019a), these effects may not be subject to same controls during the daytime as melatonin levels are minimal during the biological day (Dijk et al., 1992; Hull et al., 2003; Akerstedt et al., 2017). A relatively short duration of light exposure during the daytime might thus not be enough to elicit an alertness response. For example, a recent study explored the effects of the CCT on subjective alertness, effort-related cardiac response, and cognitive performance during the daytime. The results showed that 4 min of exposure to light did not elicit significant changes in alertness and task performance (Lasauskaite et al., 2018). Similarly, several previous studies using 15 min (Iskra-Golec and Smith, 2008) or 55 min (Huiberts et al., 2016) of light exposure revealed non-significant effects on the performance or subjective indicators of alertness. Several recent studies found that bright light did not have an effect on alertness even when the duration of exposure was extended to 1 h (Lok et al., 2018b; Smolders et al., 2018) or 90 min (Lok et al., 2019; De Vries et al., 2020). However, bright light has been shown to increase subjective alertness in the case of exposure to it for 3 h (Münch et al., 2016), 4 h (Ruger et al., 2006), and 5 h (Phipps-Nelson et al., 2003). Although the aforementioned studies suggest that different durations of bright light exposure might lead to inconsistencies in results, it is still difficult to directly compare findings in different studies given the differences in study paradigm and the various light manipulations that were employed. Studies to discern the relationship between alertness and bright light exposure in the daytime for extended durations are, therefore, warranted. Thus, current research is concerned with exploring whether extended exposure durations of up to 5 h will moderate alertness response of an individual to bright light in the afternoon. In addition, the study design seeks to address whether participants experience more or less mental fatigue after exposure. The light exposure period of 5 h served to capture how alertness dynamically changes under bright light illumination. The results of the current study could provide evidence-based data in support of healthy lighting design and intervention applications.

In addition to the duration of light exposure, the various indicators employed to assess the alertness response of an individual to light reported in previous studies make it difficult to compare their findings directly. Additionally, it should be noted that assessment of previous studies has revealed a rather inconsistent pattern concerning the effects of daytime bright light vs. dim light on the subjective and objective indicators of alertness within a similar study paradigm (Smolders et al., 2012; Huiberts et al., 2015, 2016, 2017). Studies employing subjective indicators of alertness even revealed inconsistent findings (Smolders and De Kort, 2014; Leichtfried et al., 2015; Huiberts et al., 2016; Weisgerber et al., 2017). Only four studies, which involved three indicators of alertness, yielded consistent findings (one with no effect (Badia et al., 1991) and three with positive results (Phipps-Nelson et al., 2003; Huiberts et al., 2015; Münch et al., 2016). Maierova et al. (2016) and Te Kulve et al. (2017) reported a positive effect of bright light on subjective alertness but not on objective alertness. These results suggested that the various indicators of alertness with respect to sensitivity respond differently to light manipulation during the daytime. To address this problem, the use of a multi-measurement approach might be a feasible option for directly comparing the alertness effects caused by bright light on subjective ratings, task performance, and EEG activity.

To investigate the temporal dynamics of alertness during the daytime, the present study utilized extended exposure duration periods of up to 5 h, and a multi-measurement approach was adopted to trace the trajectory effects of bright light on the subjective, objective, and physiological alertness during the afternoon. Participants were assigned to the conditions that experienced exposure to bright light vs. regular office light for 5 h during the daytime, and during which the Karolinska sleepiness scale (KSS), an auditory PVT, and the waking EEG were repeatedly measured during light manipulation. It was hypothesized that exposure to bright light vs. office light in the afternoon would induce an exposure duration-dependent alertness effect, and with more pronounced effects on alertness indicators being experienced as the duration of exposure was extended.

## Materials and Methods

### Experimental Design

A within-subject design was used in the study with the light conditions (200 lx vs. 1,200 lx at 6,500 K at eye level) being the independent factor. All participants visited the laboratory twice at intervals of at least 1 week. The participants were exposed to light from a light box for 5 consecutive hours. The order of light condition was balanced across the participants with half of the participants receiving the bright light first.

### Participants

Twenty participants (11 female, 20–30 years old, mean age 23.25 years, *SD* = 2.3) met all criteria for the laboratory-based study. The criteria for inclusion in the study were as follows: (1) no extreme chronotype according to the Munich Chronotype Questionnaire (Roenneberg et al., 2003), (2) no color blindness, (3) no diagnosis of physiological or psychological disease based on a subjective report, (4) had not traveled across time zones for a month before the commencement of the study, and (5) had had a regular sleep (7–9 h), which meant going to bed between 21:00 pm and 24:00 pm and waking up time between 6:00 am and 10:00 am. The research was approved by the National Chengchi university Ethical Committee and was conducted in accordance with the requisite university guidelines and the principles of the Helsinki Declaration.

### Room and Lighting Settings

The study was conducted in a laboratory with two separate compartmentalized rooms. The rooms were soundproofed with no additional light source. The walls and ceilings of the rooms were all white, and the floors were solid wood. The dimensions of the rooms were 5.7 m × 2.5 m × 3.1 m and 3.7 m × 2.5 m × 3.1 m, respectively. Each room had a table on which a light box had been placed. The light box was a cuboid made of wooden materials. White paint inside the box caused the light to be evenly distributed. Bright white light and common light were directed to the corneas of participants by arrays of light-emitting diodes (LEDs) to avoid direct viewing of the light. The light was emitted from a hole in the top of the box.

**FIGURE 1:**
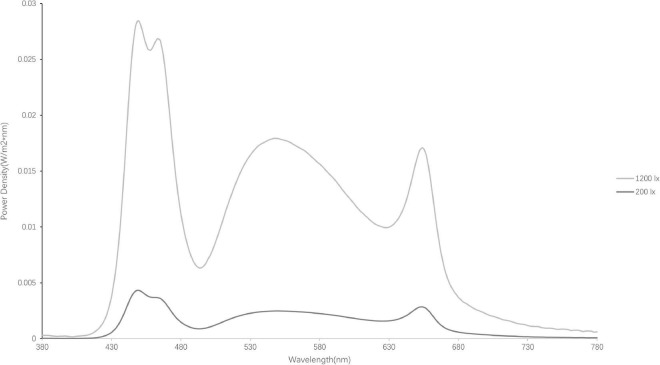
Spectral power distributions of the light sources (1,200 lx and 200 lx).

The spectral power distributions (SPD) of the two light conditions (1,200 lx at 6,500 K and 200 lx at 6,500 K) are presented in Figure 1 and were measured by a TES-1335 digital illuminance meter (TES Electrical Electronic Corp., Taipei, Taiwan) at eye level. The light level was 100 lx at 6,500 K at eye level for baseline measurement for both light conditions. Then the light level was adjusted to either 1,200 lx (photon density: 1.04 × 10^15^ photons s^–1^ cm^–2^; irradiance: 375.14 μW cm^–2^) or 200 lx (photon density: 1.54 × 10^14^ photons s^–1^ cm^–2^; irradiance: 55.30 μW cm^–2^) at 6,500 K at eye level after the baseline phase. The effective irradiances of each retinal photoreceptor provided by the two light conditions are shown in Table 1 (Lucas et al., 2014; Spitschan et al., 2019). During the experiment, the temperature in the rooms was maintained at 25°C by air conditioning.

**TABLE 1:** Spectrally weighted α-optic irradiance for each light condition.

Sensitivity	λmax (nm)	α-optic lux value	
		200 lx	1200 lx
Melanopsin	480	149.24	1058.04
S-cone	419	162.34	1111.49
M-cone	530.8	152.76	1077.13
L-cone	558.4	150.87	1044.25
Rods	496.3	150.55	1069.02
			

### Procedure

**FIGURE 2:**
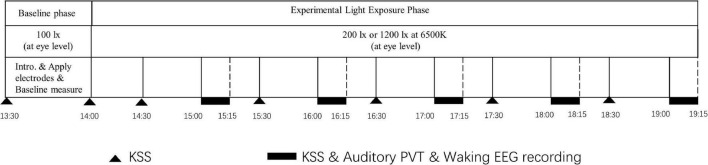
Overview of the procedural steps of the experiment.

Before commencing the study, all participants provided their informed consent and completed a screening questionnaire. Only the qualified participants were allowed to participate in the formal experiments. The laboratory sessions began at 13:00 pm and ended at 19:00 pm and included 30 min for the baseline measurement and 5 h for light exposure. An overview of the experiment procedure is given in Figure 2. During the baseline measurement, the KSS, a 10-min auditory PVT, and a 3-min waking EEG recording were conducted under 100 lx at 6,500 K (at eye level). After the baseline assessment, the light level was adjusted to either 200 lx or 1,200 lx (at 6,500 K at eye level) for 5 consecutive h (from 14:00 pm to 19:00 pm), during which time the subjective sleepiness of participants was measured every half hour (T1, T2, T3, T4, T5, T6, T7, T8, T9, and T10). The PVT and the waking EEG were assessed hourly from 14:00 pm to 19:00 pm [T2 (15:00), T4 (16:00), T6 (17:00), T8 (18:00), and T10 (19:00)].

The participants were required to maintain a fixed sitting posture in front of the light box and could not engage in activities, such as listening to music or reading to eliminate the interference of potentially interfering variables, such as activities and exposure to natural light, during the 5-h light intervention. The participants were permitted to drink water but coffee or other beverages containing caffeine and alcohol were forbidden on the days of the experiments. At about 18:00 pm, the participants were given a light meal that could be finished within about 5 min. During the EEG recording, the participants were asked to relax, keep their eyes open, and focus on a spot on the light box while avoiding frequent blinking and head movements (as shown in Figure 2 for an overview of the experimental procedure).

### Measures

#### Subjective Alertness

The KSS was used to assess subjective alertness, the values ranging from 1, “very alert,” to 9, “extremely sleepy” (Akerstedt and Gillberg, 1990). The KSS values were collected every half hour.

#### Objective Alertness

The auditory PVT was employed to assess objective alertness (Dinges and Powell, 1985). For this test, audible beeps (100 ms, 1,000 Hz, 60 dB) were delivered through a pair of headphones at randomized intervals of 2–10 s. The participants were required to press the space bar on the keyboard as soon as they heard the beep. The average reaction speed in the overall trials, the fastest 10% trials, and the slowest 10% trials were calculated separately as objective indicators of alertness.

#### Physiological Alertness

The EEG was recorded during the Karolinska drowsiness test (KDT) (Akerstedt and Gillberg, 1990) at six referential channels—C3–A2, C4–A1, F3–A2, F4–A1, O1–A2, and O2–A1—by using the Embla digital system (Embla N7000 digital PSG system; Rembrandt Embla Company, Broomfield, CO, United States). During the EEG recordings, the participants were required to sit in front of the light boxes without moving their heads, look straight ahead at a sign, and not blink or shake the body for 3 min. All EEG signals were low-pass filtered at 35 Hz and high-pass filtered at 0.3 Hz at a sampling rate of 200 Hz. All subsequent EEG data were analyzed using the Brain Product Analyzer software. The differential standard has been used to classify the EEG band in previous studies (Cajochen et al., 2005; Besserve et al., 2007; Rahman et al., 2014; Sahin et al., 2014; Iskra-Golec et al., 2017; Hsiao et al., 2018). In the current study, the classification of the EEG band, as referred to in the study of Hsiao et al. (2018), corresponds to the frequency bands were defined as delta (0.5–2.5 Hz), theta (2.5–7.5 Hz), alpha (7.5–12 Hz), and beta (14–35 Hz). Expanding the range of theta bands enables one to explore how the low-frequency theta bands vary under bright light conditions (Makeig et al., 2000). The relative ratio of the relative EEG power to the power ratio obtained at the baseline was calculated in the statistical analysis. Similar to the PVT, the EEG data were collected hourly after the PVT. Light-induced moderations of the theta, alpha, and beta bands were used as indicators of physiological alertness (Lockley et al., 2006; Phipps-Nelson et al., 2009; Cajochen et al., 2011; Rahman et al., 2014).

### Statistical Analysis

Before further analysis, the reaction speed recorded on the PVT was inverted to increase the normality of the raw data. Because of the nested structure of the data, the linear mixed model (LMM) analyses were performed to investigate the effects of light conditions on subjective alertness, task performance, and resting EEG. Given that the order for exposure by the two light conditions was balanced across the participants, the “order” of the light conditions was used as a fixed factor in the preliminary model. The results obtained did not reveal any significant effects, such as the interaction between the order and the light conditions or possible interactions between the order, light conditions, and test time. Moreover, the subjective alertness, task performance, and physiological indicators were measured multiple times for the two light conditions; and the test time was added as an additional fixed factor in the model. It was surmised that the multiple measurements may result in a correlation occurring between repeated measurements within one session thus chi-square tests were used to identify the differences in the outcomes between the models to verify whether their fitness had significantly improved (Field, 2013). AR(1) was used as a repeated covariance structure based on chi-square comparisons of the null models with different repeated covariance types [Scaled Identity, Autoregressive (1), and Diagonal].

An LMM was employed with light condition (200 lx vs. 1,200 lx) as a fixed factor and Participant ID was used as the random intercept for subjective alertness, PVT performance, and EEG activity to check the differences in the baseline measurements. Subsequently, LMM was used with light condition (200 lx vs. 1,200 lx) and test time (T1–T10) as a fixed factor, and participant ID and Session as the random intercept to evaluate the effect of light condition on the subjective alertness. The LMMs with light conditions (200 lx vs. 1,200 lx) and test time (T2, T4, T6, T8, and T10) as a fixed factor, and Participant ID and Session as the random intercept were employed to evaluate the effect of light conditions on alertness performance and resting EEG power. Effect sizes (one of the pseudo *R*^2^-values indicator $R_{\text{L}}^{2}$) were calculated by dividing the model chi-square, which represents the change from the null model by the null model—2log-likelihood. $R_{\text{L}}^{2}$ is a measure of the proportional reduction in the absolute value of the log-likelihood value, which is a determinant of the extent to which the fit improves as a result of the inclusion of the predictor variables (Field, 2013). The change in value is from 0 to 1, where 0 represents the condition whereby the predictors are unable to predict the outcome variable and 1 means that the model predicts the outcome variable perfectly (Hosmer and Lemeshow, 2013). The *post-hoc* tests with the Bonferroni correction were used to investigate differences in the dependent variables of the test time, the estimated marginal means (EMMs), and the SEs. All the statistical analyses were performed using SPSS Statistics 25.

## Results

### Baseline Comparisons

The LMM analyses of the KSS scores and performance on the PVT at the baseline revealed no significant difference between light conditions (*p* > 0.1). However, significant differences were found in several spectral bands in different channels between light conditions at the baseline. Thus, all waking EEG data were processed by dividing the original data for T2, T4, T6, T8, and T10 by the baseline T0. The original data were normalized before further statistical analysis.

### Effects of Light Condition on Alertness

#### Subjective Alertness (Karolinska Sleepiness Scale)

**FIGURE 3:**
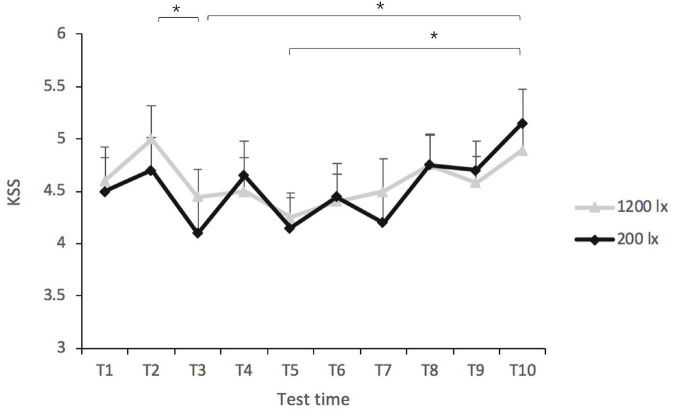
Scores of the Karolinska sleepiness scale (KSS) in the 200 lx (dim) and 1,200 lx (bright) conditions. *p < 0.05.

The LMM analyses of the KSS scores revealed no significant effect of light condition [*F*_(1, 64)_ = 0.188, *p* = 0.666], nor that of light condition × test time interaction [*F*_(9, 220)_ = 0.521, *p* = 0.858]. Test time did have a significant main effect [*F*_(9, 232)_ = 3.68, *p* < 0.001, *R*^2p*seudo*^ = 0.032] (as shown in Figure 3). The *post-hoc* comparisons indicated that the KSS scores at T3 (*EMM*_T3_ = 4.28, *SE* = 0.24) and T5 (*EMM*_T5_ = 4.20, *SE* = 0.24) were both significantly lower than that at T10 (*EMM*_T10_ = 5.04, *SE* = 0.24, all *p* < 0.05), suggesting that the participants felt more alert after 1.5 and 2.5 h of light exposure than at the end of the 5-h exposure. The KSS score at T2 (*EMM*_T2_ = 4.86, *SE* = 0.24) was significantly higher than that at T3 (*EMM*_T3_ = 4.28, *SE* = 0.24, *p* = 0.045), suggesting that the participants felt more alert after 1.5 h of exposure than after 1 h.

#### Objective Alertness (Psychomotor Vigilance Task)

**FIGURE 4:**
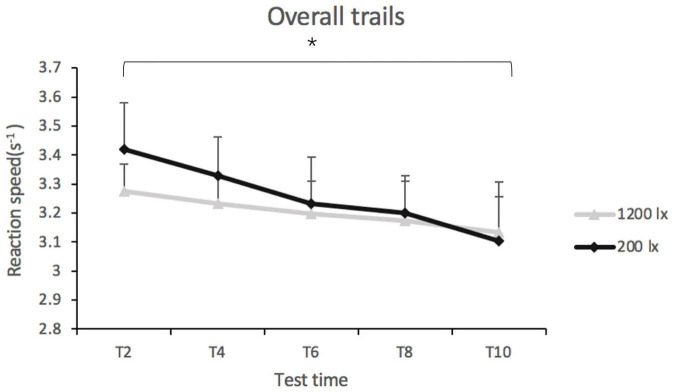
Overall reaction speed in trials of psychomotor vigilance test (PVT) in 200 lx (dim) and 1,200 lx (bright) conditions. *p < 0.05.

Light condition [*F*_(1, 41)_ = 0.77, *p* = 0.384] and light condition × test time had no significant effect ([*F*_(1, 137)_ = 0.25, *p* = 0.911] on the overall reaction speed on the PVT. Test time did have a significant main effect on the overall reaction speed [*F*_(4, 139)_ = 2.86, *p* < 0.05, *R*^2pseudo^ = 0.160] (as shown in Figure 4). The *post-hoc* tests indicated that the reaction speed was faster at T2 (*EMM*_T2_ = 3.34, *SE* = 0.13) than at T10 (*EMM*_T10_ = 3.12, *SE* = 0.13), suggesting that the reaction time exhibited an increasing trend with time (as shown in Figure 4). The estimates of reaction speed at T4 (*EMM*_T4_ = 3.27, *SE* = 0.13), T6 (*EMM*_T6_ = 3.21, *SE* = 0.13), and T8 (*EMM*_T8_ = 3.18, *SE* = 0.13) were not significantly different.

The LMM analysis of the fastest 10% of reaction speeds revealed no significant effect of light condition [*F*_(1, 36)_ = 2.35, *p* = 0.134], test time [*F*_(4, 137)_ = 1.89, *p* = 0.116], or that due to the interaction between light condition × test time [*F*_(4, 122)_ = 0.089, *p* = 0.986]. Similarly, light condition, test time, and the interaction between them had no significant effect on the slowest 10% of reaction speeds [*F*_(1, 40)_ = 0.07, *p* = 0.794; *F*_(4, 139)_ = 2.23, *p* = 0.069; and *F*_(4, 128)_ = 0.66, *p* = 0.621].

#### Physiological Alertness (Electroencephalogram)

**FIGURE 5:**
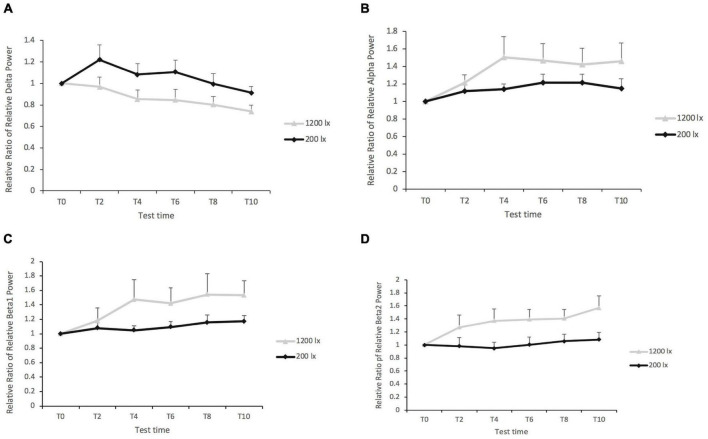
**(A)** The relative ratio of the relative delta power in region F3 between 1,200 and 200 lx. **(B)** The relative ratio of the relative alpha power in region O1 between 1,200 and 200 lx. **(C)** The relative ratio of the relative beta1 power in region F3 between 1,200 and 200 lx. **(D)** The relative ratio of relative beta2 power in region F3 between 1,200 and 200 lx. The figure shows only some of the significant results of the channels.

Light condition had a significant main effect on the delta band at F3 [*F*_(1, 42)_ = 8.46, *p* = 0.006, *R*^2pseudo^ = 0.157], F4 [*F*_(1, 78)_ = 7.86, *p* = 0.006, *R*^2pseudo^ = 0.012], C3 [*F*_(1, 48)_ = 5.79, *p* = 0.020, *R*^2pseudo^ = 0.05], and O2 [*F*_(1, 45)_ = 5.12, *p* = 0.028, *R*^2pseudo^ = 0.057]. The *post-hoc* tests revealed a significantly lower delta band under 1,200 lx condition (*EMM*_1_,_200__*lx*_ = 0.84; *SE* = 0.08) compared with the 200 lx exposure (*EMM*_200__*lx*_ = 1.06; *SE* = 0.08) at F3 (as shown in Figure 5A); the same results were noted on channels F4, C3, and O2, with lower delta bands being observed for the 1,200 lx condition compared with the 200 lx condition. The effect of test time were also significant on the delta band at channels F3 [*F*_(4, 154)_ = 3.36, *p* = 0.012] and F4 [*F*_(4, 154)_ = 2.83, *p* = 0.027]. The *post-hoc* tests showed that the delta band was significantly higher for test time 2 (*EMM*_T2_ = 1.09; *SE* = 0.07) compared with test time 10 (*EMM*_T10_ = 0.83; *SE* = 0.07) at F3. At channel F4, the delta band was significantly higher for test time 2 (*EMM*_T2_ = 1.11; *SE* = 0.07) compared with test time 10 (*EMM*_T10_ = 0.84; *SE* = 0.07). No significant difference was found in the delta band at C4 [*F*_(1, 53)_ = 2.99, *p* = 0.089] and O1 [*F*_(1, 38)_ = 1.28, *p* = 0.265]. There was no significant interaction effect (all *F < 1*, *p’s* > 0.05) on the delta band either.

The results for the theta band revealed no significant main effects or interaction effects (all *p* > 0.05).

In the case of the alpha band, the results revealed a significant main effect for the light condition at channel O1 [*F*_(1, 101)_ = 4.03, *p* = 0.047, *R*^2pseudo^ = 0.06], with a higher alpha band in the 1,200 lx condition than in the 200 lx condition (as shown in Figure 5B). Light condition elicited no significant difference in the alpha power spectrum in the central and frontal areas of the brain (all *p* > 0.05). The main effect of test time (all *p* > 0.05) and its interaction effects (all *ps* > 0.05) did not have significance on the alpha band.

Light condition had significant effects on both the beta1 (as shown in Figure 5C) and the beta2 bands (as shown in Figure 5D) on all channels (all *p* < 0.05), except for the beta1 band on C4 (*p* = 0.066). The *post-hoc* tests revealed higher beta1 and beta2 for the 1,200 lx condition than the 200 lx light condition. The main effect of test time, and the interaction between the light condition and the test time, was not significant on the powers of beta1 and beta2 (all *p* > 0.05).

## Discussion

The effects of exposure to bright light versus office light (1,200 vs. 200 lx at eye level, 6,500 K) for 5 h in the afternoon on the alertness of participants were examined using a multi-measurement experimental design strategy. The results revealed that prolonged exposure to a higher level of light in the afternoon lowered the relative delta power and increased the relative beta power in comparison with exposure to regular office light, indicating an increase in cortical arousal. However, the subjective measure of alertness (KSS) and performance on the vigilance test (PVT) remained unaffected as a result of light manipulation.

The findings revealed that 5 h of exposure to 1,200 vs. 200 lx at 6,500 K light in the afternoon elicited no statistically significant improvement of subjective alertness as assessed with the KSS, and this is consistent with the previous studies suggesting no significant effects of bright light vs. dim light on subjective alertness for relatively short (i.e., 55 min) or longer durations (i.e., 110 min) (Sahin et al., 2014; Huiberts et al., 2016; Lok et al., 2018b; Smolders et al., 2018; De Vries et al., 2020). In contrast, other studies have reported significant effects from exposure to bright light for 90 min–5 h during the daytime (Phipps-Nelson et al., 2003; Ruger et al., 2006; Maierova et al., 2016; Münch et al., 2016). Such discrepancies might have occurred due to the difference in light levels in the experimental sessions or the baseline measurements (ranging from ≤ 10–40 lx vs. 200 lx for the present study) (Akerstedt et al., 2003; Phipps-Nelson et al., 2003; Ruger et al., 2006; Vandewalle et al., 2006; Sahin et al., 2014; Maierova et al., 2016; Münch et al., 2016; Te Kulve et al., 2017). Additionally, the monotony of remaining for 5 h in front of a lamp without performing any tasks is probably the reason for finding no significant results related to subjective alertness. Moreover, the participants did feel more alert after 1.5 and 2.5 h of light exposure (T3 and T5) than at the end of the experimental session (T10) for both light conditions, indicating that participants did felt significantly less alert at the end of the 5 h session than earlier in the session regardless of the light conditions. We thus speculate that when participants lack any activities and are under exposure to 1,200 lx vs. 200 lx blue-enriched white light for 5 h, it is not possible to prevent a decline in subjective alertness over 5 h in the afternoon.

In addition to subjective alertness, the findings revealed that exposure to 5 h of 1,200 lx vs. 200 lx light exposure had no significant effect on the performance in terms of the overall response speed, or the fastest 10% or slowest 10% of reaction speeds in the PVT. The present results replicate certain previous findings (Huiberts et al., 2016; Maierova et al., 2016; Te Kulve et al., 2017; Smolders et al., 2018; Lok et al., 2019) while contradicting others (Phipps-Nelson et al., 2003; Smolders et al., 2012; Smolders and De Kort, 2014; Münch et al., 2016; Huiberts et al., 2017). These inconsistencies in the results of independent studies might reflect differences in methodology. For instance, the participants were sleep-restricted in the study by Phipps-Nelson et al. (2003), and the participants were subjected to mental fatigue before light manipulation in the study of Smolders and De Kort (2014). In contrast, the participants in the current study were all well-rested. Moreover, the variability in illumination parameters and exposure durations used in previous studies may partly explain the discrepancies. Münch et al. (2016) explored the influence of light of 750 lx vs. 40 lx at eye level for 3 h for 3 days, whereas exposure to 1,200 lx and 200 lx light for 5 h was adopted in the present study. The results of previous studies suggested a lasting and accumulative effect of daytime light on the later days (Gabel et al., 2015) or employing a relatively dimmer light (i.e., 40 lx Münch et al., 2016) as a control condition, although such a dim light level would not be typical for what office workers experienced in real-work situations. Besides, some previous studies have reported a time-of-day dependent alertness effect of bright light during the daytime (Smolders et al., 2012; Huiberts et al., 2017), with more pronounced effects on PVT performance in the morning than in the afternoon. However, whether the alertness effect of 5-h bright light exposure occurs for exposure in the morning remains unknown. Except for the insignificant differences in the PVT between the light conditions, the current findings revealed that the overall reaction speed in the PVT decreased at the end of the experimental session, which corresponded to an increasing trend in subjective sleepiness as indicated by the KSS scores. These effects might have occurred owing to an accumulated pressure for sleep as the waking time increased (Borbely, 1982; Borbely et al., 2016).

The theta, alpha, and beta bands were investigated as indicators of psychophysiological alertness (Santamaria and Chiappa, 1987; Aeschbach et al., 1999; Craig et al., 2012; Ishii et al., 2014). With this, the increased high-frequency spectral power (i.e., of the beta bands) and the decreased low-frequency spectral power (i.e., of the theta-alpha and theta bands) indicate higher physical alertness and arousal in the case of the former (Badia et al., 1991; Aeschbach et al., 1999; Strijkstra et al., 2003; Kaida et al., 2006a; Lockley et al., 2006; Phipps-Nelson et al., 2009; Cajochen et al., 2011; Craig et al., 2012; Rahman et al., 2014). The current results revealed that the ratio of the alpha band was significantly more for the 1,200 lx vs. 200 lx condition at O1, and the ratio of the beta band was significantly more for the 1,200 vs. 200 lx condition in all brain regions except the C4 channel. These findings indicated that exposure to 1,200 lx vs. 200 lx light moderated EEG activities somewhat differently across the spectral bands. Only three studies as reviewed by Lok et al. (2018a) assessed the effects of light exposure on the EEG-based beta activity during the daytime. None of these studies have reported a significant light-induced regularity effect on EEG activity (Badia et al., 1991; Sahin et al., 2014; Smolders et al., 2016). However, the comparative exposure levels increased both the relative beta1 and beta2 powers. In addition to the differences in the properties of the light, the multiple measurement strategy employed in this study may facilitate detection in view of the light-induced moderation of neurophysiological activity. However, the results reveal an insignificant effect of the light level on the power of theta, which is consistent with the results of certain previous studies (Badia et al., 1991; Akerstedt et al., 2003; Smolders et al., 2016) while contradicting the findings of others (Kaida et al., 2006a; Sahin et al., 2014). As mentioned above, the difference in the properties of light makes it difficult to directly compare present findings with those of independent studies. For instance, natural light (Kaida et al., 2006b) or dim light (<5 lx) was used as the control condition employed in the study of Sahin et al. (2014), while LED light (200 lx) was used as the control light condition in the present study. Most previous studies have not examined the delta band or reported the moderation of delta activity by light (Badia et al., 1991; Akerstedt et al., 2003; Sahin et al., 2014; Smolders et al., 2016) which has been documented mainly as an indicator of homeostatic sleep pressure. In the current study, the EEG delta activity decreased for 1,200 vs. 200 lx light in the afternoon. One earlier study also reported that daytime bright light (1,700 lx vs. 450 lx at the desk) during the daytime decreased the delta activity. It was speculated by Küller and Wetterberg (1993) that bright light might cause an alertness effect on the central nervous system. Iskra-Golec et al. (2017) investigated the effect of blue light on EEG activity in the daytime and found that the delta band decreased under blue light; it was concluded that decreases in the delta wave were correlated negatively with subjective sleepiness and fatigue (Lal and Craig, 2002).

The moderating effect of light on neural activity in different regions of the brain was examined. The results revealed that the delta band was lower for 1,200 lx light compared with 200 lx light for all channels except C4 (*p* = 0.085) and O1 (*p* = 0.437), indicating that the bright light-induced moderation of the delta band was subtly affected by these regions. A similar situation was observed for the beta1 and beta2 bands, with increased beta in the 1,200 lx vs. 200 lx condition for all channels except beta1 at C4 (*p* = 0.066). However, the relative power density of the theta band remained unaffected by the light condition for all channels. For the alpha band, the findings showed that the 1,200 lx vs. 200 lx condition elicited a higher density for the alpha band but only in the occipital region, which is consistent in part with the previous studies (Badia et al., 1991; Akerstedt et al., 2003; Smolders et al., 2016) while at the same time contradicting other research (Kaida et al., 2006b; Sahin et al., 2014). One factor which might contribute to these discrepancies is the differential analysis of the strategies considered. For example, the power density of a single channel (Kaida et al., 2006b; Sahin et al., 2014) or the average power density of multiple channels (Sahin et al., 2014; Smolders et al., 2016) were used as the dependent variable for EEG activity, which limited the ability to detect regional differences between the light conditions. Together, these findings suggest that bright light vs. the regular office light elicits a localization- and spectrum-dependent moderation of neurophysiological activities in the brain.

The current findings, however, did not reveal a consistent pattern that emerged between the effect of bright light on the subjective, objective, and physiological alertness. Both the subjective alertness and the vigilance performance were shown to decrease over time regardless of the light condition, however, the physiological alertness, as assessed by using the EEG power, was significantly influenced by the light condition. These findings suggest that the EEG activity might be more sensitive to the bright light induced alertness response during the daytime compared with the subjective ratings and task performance. These findings were partly in line with previous studies concerned with assessing the alertness effects of bright vs. dim light using multiple measures of alertness as described by Te Kulve et al. (2017). Nevertheless, the differences in exposure duration and time of day made it difficult to directly compare the findings of previous studies and present research. Previous studies suggested that the consistency of subjective and objective indicators would be affected by the degree of sleep deprivation, the experimental design (within or between subjects), and statistical approaches taken to compare associations across time (Zhou et al., 2012; Mulhall et al., 2019; Manousakis et al., 2021). Thus, for the moment, we can suggest that multiple measures of alertness are necessary for assessing the alertness effect of daytime light in other experimental scenarios (e.g., in the morning or the entire day) in the future.

The results of this study should be treated with some caution owing to several limitations. First, the early evening period (18:00 pm–19:00 pm) was included in the experimental session, where such endogenous factors as hormonal melatonin, although not measured here, should not be neglected. Second, the time-of-day effects of bright vs. dim light during daytime have been reported in several previous studies (Ruger et al., 2006; Smolders et al., 2012; Huiberts et al., 2015, 2017). This study was conducted only in the afternoon; hence the findings may not be transferrable to other times of the day. Third, some variables were not well controlled in this study, such as the light exposure history and sleep quality of the participants before commencing the laboratory study. Although the participants were asked to maintain a regular sleep routine and keep away from sunlight as much as possible before the test, the contribution of these additional variables cannot be ruled out. Fourth, lack of control of food intake at lunch on the experiment days leads to another limitation, which may affect the participant’s state of alertness in the afternoon. Last, the tasks used in the current study were rather monotonous, repetitive, and the lack of activities during the light intervention made it difficult for participants to stay awake. Such a situation would not reflect the activities of an actual workday for the participants. More experimental work is needed to evaluate the generalization of current findings.

## Conclusion

The results of the current study revealed that, in general, prolonged exposure to bright light in the afternoon moderates the physiological level of arousal as measured by EEG activity; however, no statistically significant change in subjective ratings and performance measures of alertness were observed. Exposure to a blue-enriched white light (1,200 lx, 6,500 K) vs. regular office light (200 lx, 6,500 K) significantly lowered the relative delta power and increased the relative beta power. These findings suggest that exposure to bright light for 5 h in the afternoon compared with regular office light level enhances physiological arousal levels in terms of the delta power and beta power but at the same time exerts no influence on subjective alertness or performance with respect to vigilance.

## Data Availability Statement

The original contributions presented in the study are included in the article/supplementary material, further inquiries can be directed to the corresponding author/s.

## Ethics Statement

The studies involving human participants were reviewed and approved by the Ethical Committee of National Chengchi University. The patients/participants provided their written informed consent to participate in this study.

## Author Contributions

XL: conceptualization, methodology, software, investigation, formal analysis, writing and original draft preparation, writing, reviewing, and editing. TR: supervision, conceptualization, methodology, validation, writing, reviewing, and editing. QC: data curation and visualization. F-CH and C-SH: subjects recruitment and clinical assessment. C-MY: conceptualization and methodology. GZ: project administration and funding acquisition. All authors reviewed the manuscript.

## Conflict of Interest

The authors declare that the research was conducted in the absence of any commercial or financial relationships that could be construed as a potential conflict of interest.

## Publisher’s Note

All claims expressed in this article are solely those of the authors and do not necessarily represent those of their affiliated organizations, or those of the publisher, the editors and the reviewers. Any product that may be evaluated in this article, or claim that may be made by its manufacturer, is not guaranteed or endorsed by the publisher.
